# Release behaviour and toxicity evaluation of levodopa from carboxylated single-walled carbon nanotubes

**DOI:** 10.3762/bjnano.6.23

**Published:** 2015-01-22

**Authors:** Julia M Tan, Jhi Biau Foo, Sharida Fakurazi, Mohd Zobir Hussein

**Affiliations:** 1 Materials Synthesis and Characterization Laboratory, Institute of Advanced Technology (ITMA), Universiti Putra Malaysia, 43400 UPM Serdang, Selangor, Malaysia; 2 Laboratory of Molecular Biomedicine, Institute of Bioscience (IBS), Universiti Putra Malaysia, 43400 UPM Serdang, Selangor, Malaysia; 3 Laboratory of Vaccine and Immunotherapeutics, Institute of Bioscience (IBS), Universiti Putra Malaysia, 43400 UPM Serdang, Selangor, Malaysia,; 4 Department of Human Anatomy, Faculty of Medicine and Health Sciences, Universiti Putra Malaysia, 43400 UPM Serdang, Selangor, Malaysia

**Keywords:** carboxylic acid-functionalized single-walled carbon nanotubes, levodopa, MTT assay, nanomedicine, Parkinson’s disease, PC12 cells, sustained release

## Abstract

This work explores the potential use of commercially obtained, carboxylated, single-walled carbon nanotubes (SWCNT–COOH) as nanocarriers for the antiparkinson drug, levodopa (LD). The resulting nanohybrid was characterized using materials characterization methods including Fourier transform infrared spectroscopy, Raman spectroscopy, elemental analysis, UV–vis spectroscopy and scanning electron microscopy. The results showed that SWCNT–COOH were able to form supramolecular complexes with LD via a π–π stacking interaction and exhibited favourable, slow, sustained-release characteristics as a drug carrier with a release period over more than 20 h. The results obtained from the drug release studies of LD at different pH values showed that the LD-loaded nanohybrid is pH activated. The release kinetics of LD from SWCNT–COOH were well-described by a pseudo-second-order kinetic model. A cytotoxicity assay of the synthesized nanohybrid was also carried out in PC12 cell lines (a widely used, in vitro Parkinson’s model for neurotoxicity studies) using 3-(4,5-dimethylthiazol-2-yl)-2,5-diphenyltetrazolium bromide (MTT) assay in order to investigate their possible effects on normal neuronal cells in vitro. It was found that the synthesized nanohybrid did not compromise the cell viability and the PC12 cells remained stable throughout the experiments up to 72 h after treatment.

## Introduction

Over the past few years, the revolutionary development of nanomedicine has emerged as one of the most prominent research areas in biomedical science. This interdisciplinary technology is a combination of both traditional medical technology and nanotechnology, with the exploitation of nanosized materials of dimensions less than 100 nm. One such nanomedical approach to drug delivery technology that has made a great impact was the first demonstration utilizing liposomes as drug carriers for proteins and pharmaceuticals to treat diseases by Bangham and Horne in the 1960s [1]. Since then, multidisciplinary researchers have been actively investigating advanced drug delivery systems by directing drugs and/or carriers with sustained release properties directly to a the specific site of the diseased cells. Generally, drug carriers can be categorized into four major groups: inorganic nanoparticles [2–3], recombinant proteins [4], viral or non-viral carriers [5] and organic cationic compounds [6].

Recently, inorganic nanoparticles such as carbon nanotubes (CNTs) were subjected to intense research for theranostic delivery systems, especially in the field of cancer chemotherapy [7–9]. Their attractive properties such as good biocompatibility and excellent chemical and thermal stability ensure the stability and solubility of drugs in aqueous environments. Furthermore, their ultrahigh surface area can enhance the loading capacity of different macromolecules or bioactive compounds, which are chemically attached to their side walls, tips or encapsulated inside the tubes. In addition, sufficiently functionalized CNTs can also adequately reduce the cytotoxic side effects of CNTs, and at the same time, further enhance their degree of biocompatibility [7]. This is because non-functionalized CNTs tend to aggregate into bundles due to van der Waals interactions and hence, they might induce apoptosis (cell death) after administration into the human body.

Parkinson’s disease (PD) or *Paralysis Agitans* is a type of neurodegenerative disorder that affects one in every 100 persons above the average age of 65 years [10]. This disease, which affects the central nervous system, was first reported by Dr. James Parkinson in 1817 and was documented as “An Essay on the Shaking Palsy” [11]. A person diagnosed with PD shows typical motor symptoms such as resting tremor, spasticity, unstable posture, walking difficulty, dementia, slowness of body movements (bradykinesia) and involuntary movements (dyskinesia). This is due to depletion of dopamine (a catecholamine neurotransmitter) in the brain. The currently available medications are designed with the aim to improve the functional capacity of the patient for an extended period, but not on the modification of the neurodegenerative process [10].

Even though there are several antiparkinson medications available in the market, (2S)-2-amino-3-(3,4-dihydroxyphenyl)propanoic acid (levodopa) still remains the gold standard for the treatment of PD for symptomatic relief [12]. Now entering into its fourth decade of clinical use, levodopa (LD, the amino acid precursor of dopamine) is the most effective, widely prescribed, oral administered drug, due to its ability to cross the blood–brain barrier. However, responsive patients treated long term with LD therapy may experience a decrease in the duration of responsiveness to the treatment and side effects in motor fluctuation (dyskinesia) may result [13]. Moreover, once LD is administered orally into the body, the drug is immediately metabolized and only a small amount of drug reaches the central nervous system. To prevent LD from being rapidly metabolized before it reaches the brain, carbidopa, an inhibitor of dopamine decarboxylase, is commonly used in combination with LD to enhance the effectiveness of LD [14]. Therefore, in order to achieve the desired effect with a lower therapeutic dose of LD, one must take a combination of several medications, which further increase inconveniences patients.

Considering the above mentioned advantages of CNTs and the challenges exhibited by the drug itself, we describe here the synthesis of a new nanohybrid, SWCNT–LD. The objective of this research was to explore the potential use of SWCNTs for the delivery of the antiparkinson drug LD. Commercially obtained SWCNTs functionalized with carboxylic acid (–COOH) were used in this study as the starting material in order to eliminate the problems associated with the low solubility of SWCNTs [15]. We then further investigated the chemical interaction between SWCNT–COOH and LD and observed the release kinetic behaviour of LD from SWCNT–COOH. Cytotoxicity assays of the synthesized nanohybrid were also carried out in PC12 cell lines in order to evaluate their possible effects in normal neuronal cells in vitro. The results obtained from this preliminary study are expected to provide a theoretical basis and understanding for preparation of efficient drug carriers in the future.

## Results and Discussion

### Characterization of SWCNT–LD nanohybrid

**Figure 1:**
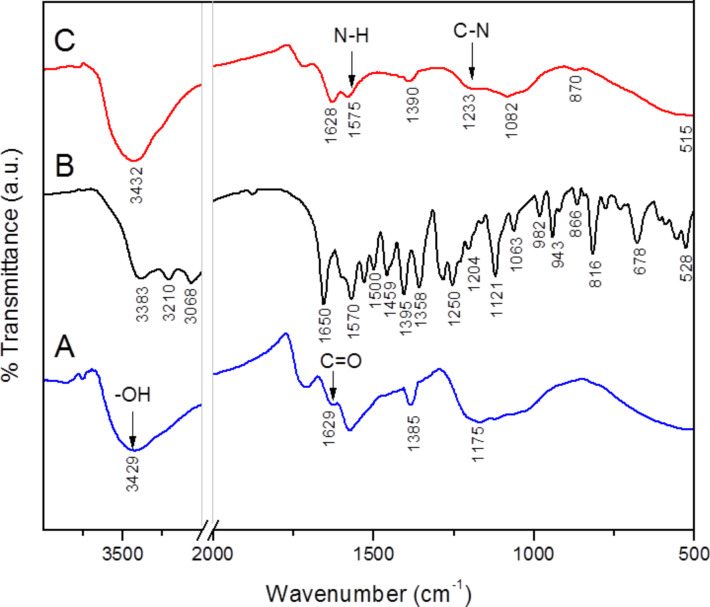
FTIR spectra of (A) SWCNT–COOH, (B) LD and (C) SWCNT–LD nanohybrid.

In addition to the supernatant residue, the solid sample nanohybrid, SWCNT–LD, was also investigated using FTIR. The FTIR spectra of the as-received SWCNT–COOH, free drug and SWCNT–LD are presented in Figure 1. The characteristic absorption peaks are observed at 3429 and 1629 cm^−1^, confirming the presence of the O–H stretching band [16] and the COO^−^ asymmetric stretching band [17], respectively, on the surface of the as-received SWCNT–COOH (Figure 1A). The FTIR spectrum of pure LD shows a number of characteristic bands at 3383, 3210, 3068, 1650, 1590, 1500, 1459, 1395, 1358, 1250, 1204, 1121, 1063, 982, 943, 866, 816, 678 and 528 cm^−1^ (Figure 1B). The bands between 3383 and 3068 cm^−1^ can be assigned to the –OH (hydroxy group) stretch in the phenol, –COOH stretch and N–H stretch vibration. A broad absorption band observed at 1650–1395 cm^−1^ is due to the aromatic rings and another band at 1250–678 cm^−1^ is due to the C–H aromatic stretching. Figure 1C shows the FTIR spectrum of the synthesized SWCNT–LD nanohybrid exhibiting the characteristic absorption bands of the two compounds. This indicates that the LD has been successfully conjugated to the SWCNT–COOH. The peak at 1575 cm^−1^ in Figure 1C is slightly shifted in position as compared to 1570 cm^−1^ in Figure 1B due to the deformation in-plane N–H bending vibration in secondary amides of the amine group. The C–N stretch appears in the spectrum of the SWCNT–LD conjugate at 1233 cm^−1^, indicating the vibrational interaction between the C–N and N–H stretching band.

The conjugation between LD and SWCNT–COOH can also be verified by elemental analysis, as shown in Table 1. As expected, the synthesized SWCNT–LD nanohybrid contained both organic (from LD) and inorganic (from the nanotubes) constituents. From the obtained CHNS results, the nanohybrid is comprised of 87.1% carbon (w/w %) and 0.7% (w/w %) hydrogen, indicating a loading of approximately 8.9% LD in the compound.

**Table 1:** Results of the CHNS elemental analysis of the SWCNT–COOH, LD and SWCNT–LD nanohybrid.

Compounds	C, [%]	H, [%]	N, [%]	SWCNT–COOH, [w/w %]	LD^a^, [w/w %]

SWCNT–COOH	86.7	0.7	–	–	–
LD	54.7	5.6	6.7	–	–
SWCNT–LD	87.1	0.7	0.6	91.1	8.9

^a^Estimated value from CHNS analysis using N (%).

**Figure 2:**
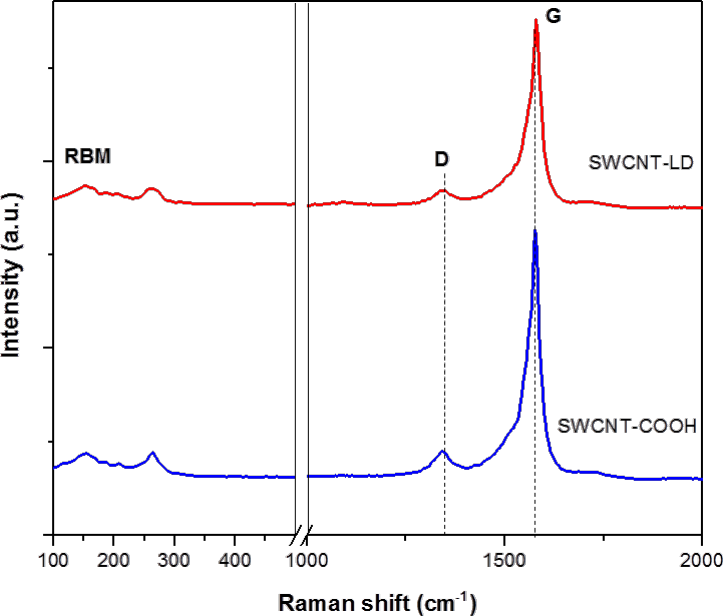
Raman spectra of the SWCNT–COOH nanocarrier and the SWCNT–LD nanohybrid.

The structural changes in the SWCNT samples before and after loading of LD were investigated using Raman spectroscopy and presented in Figure 2. All of the spectra revealed the presence of a radial breathing mode (RBM) and two characteristic bands of SWCNTs: the D-band (disorder-induced mode) was observed at 1342 cm^−1^ and the G-band (graphitic-like mode) was displayed at 1575 cm^−1^ for SWCNT–COOH and 1579 cm^−1^ for SWCNT–LD [18]. The RBM of the SWCNTs is a low frequency mode generated by the synchronous movement of the carbon atoms in the radial direction [19] and can be observed at 155 and 264 cm^−1^. The D-band is attributed to the non-crystalline quality of the carbon structures, due to defects or disorder content in the CNTs [20], whereas the G-band correlates to a high degree of ordering of crystalline graphitic structures in the CNTs (ascribed to the C–C stretching vibrations) [21]. The *I*_D_/*I*_G_ ratio for the respective D- and G-band is commonly used for qualitative analysis regarding the formation of defects in CNTs [22]. The relative intensity ratio of *I*_D_/*I*_G_ represents the degree of CNT functionalization [22], whereby a higher *I*_D_/*I*_G_ indicates a higher degree of functionalization. The D-band and G-band location and the ratio of the SWCNT samples are shown in Table 2. The ratio of *I*_D_/*I*_G_ increased from 0.273 for SWCNT–COOH to 0.310 for SWCNT–LD, which reflects the successful conjugation of LD onto SWCNT–COOH.

**Table 2:** Location and relative intensity of the Raman peaks of SWCNT–COOH and SWCNT–LD nanohybrid.

Compounds	D-band position, [cm^−1^]	G-band position, [cm^−1^]	*I*_D_/*I*_G_ intensity ratio

SWCNT–COOH	1342	1575	0.273
SWCNT–LD	1342	1579	0.310

**Figure 3:**
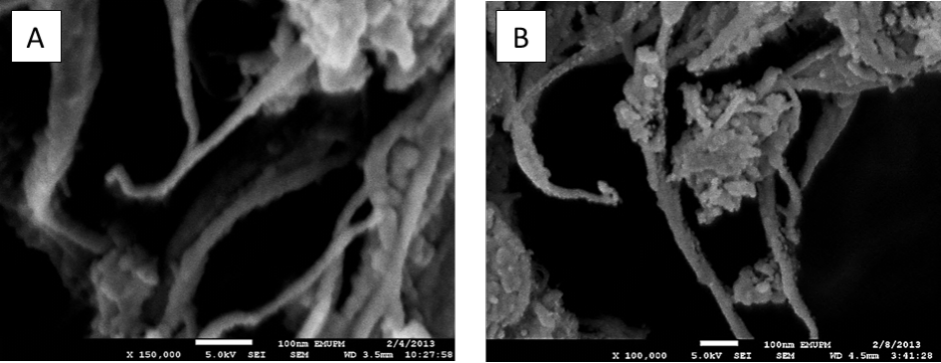
Field emission scanning electron microscopy (FESEM) images of (A) SWCNT–COOH and (B) SWCNT–LD nanohybrid.

**Figure 4:**
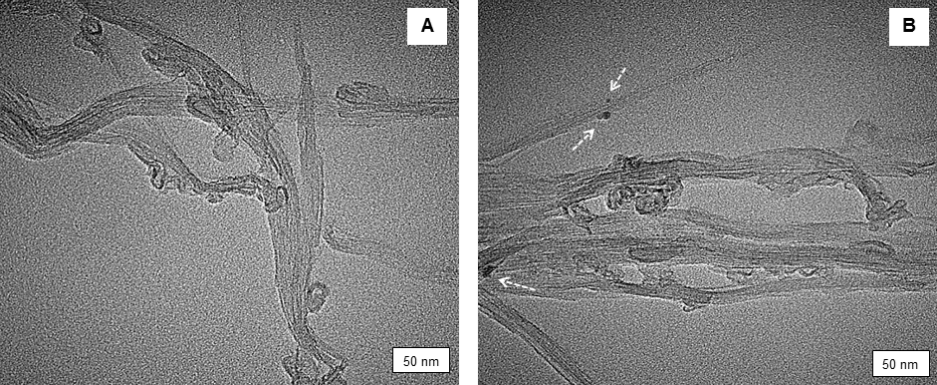
Transmission electron microscopy (TEM) micrographs of (A) SWCNT–COOH and (B) SWCNT–LD.

The morphology of the synthesized SWCNT–LD nanohybrid was further studied by field emission scanning electron microscopy (FESEM), with results presented in Figure 3. The FESEM images reveal the characteristic, tubular features of the CNTs which have a smooth surface before conjugation (Figure 3A). This structure was further investigated after coating with LD, as presented in Figure 3B, which suggests that the conjugation process had taken place. In general, the changes in Figure 3B are fundamentally different from those of the starting material shown in Figure 3A (whereby the nanotube surface is relatively clean and smooth). The FESEM images were consistent with the images obtained by transmission electron microscope (TEM), as presented in Figure 4. The internal structure of SWCNT–COOH appeared to be free from metallic impurities (Figure 4A) and after loading with LD (Figure 4B), several black spots were detected, indicating the possible locations of LD attached to the outer surface of SWCNT–COOH. A similar observation was also reported by Bhirde and coworkers using scanning TEM to detect the attachment of cisplatin (anticancer drug) onto the epidermal growth factor functionalized SWCNT [23].

### Release behaviour of LD

**Figure 5:**
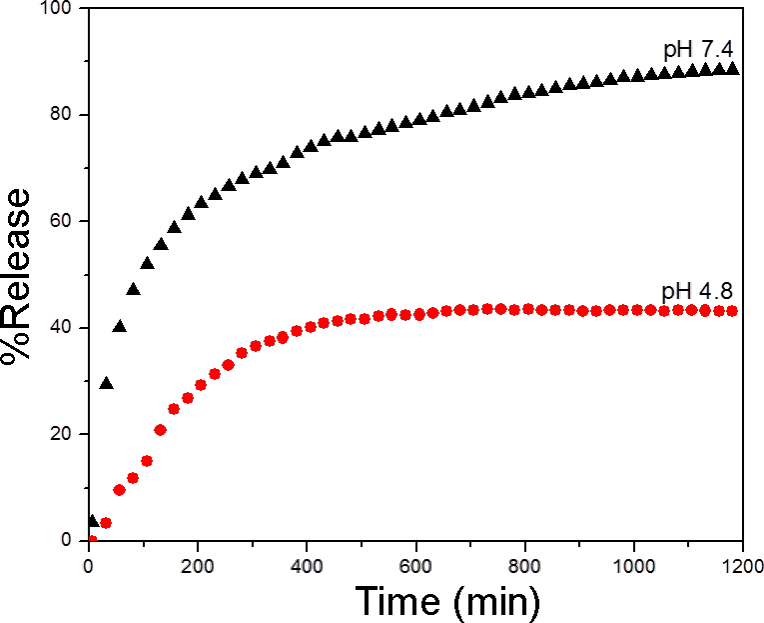
Release profiles of LD from SWCNT–COOH into phosphate-buffered saline solutions at pH 7.4 and pH 4.8.

In this study, the release profiles for LD from SWCNT–COOH at PBS pH values of 7.4 and 4.8 were investigated and shown in Figure 5. Both of the release curves show a fast release in the early stage, followed by a slower slope indicating a sustained release process, which seems to continue for a prolonged period of time. The release rate of LD at pH 7.4 was found to be significantly higher than that at pH 4.8. This indicates that the release of LD from the nanohybrid is pH-dependent. The amount of LD released from the nanohybrid reached 88.6% after 20 hours when exposed to pH 7.4 PBS solution. When the buffer was changed to a lower pH value of 4.8, the release rate of LD decreased to approximately 43.3%. This result could be mainly attributed to the protonation and deprotonation of –COOH functional groups from carboxylated CNTs. In this case, the solubility of the nanocarrier increases with an increase in pH value [24], resulting in more carboxylate anions (–COO^−^) produced in pH 7.4 as compared to pH 4.8. In addition, the different release mechanisms of LD at different pH levels could be also due to the repulsive forces between the ionized LD^−^ and SWCNT–COO^−^. LD is a drug characterized by its short, pharmacological half-life of approximately 60–90 min [25]. Therefore, the pH-sensitive, slow-release behaviour of LD with a release time of more than 20 h could reduce fluctuations in the therapeutic effect [26] and may benefit the treatment of PD.

### Release kinetics of LD

To further analyse the release kinetics of LD from the SWCNT–COOH nanocarrier, pseudo-first-order (Equation 1), pseudo-second-order (Equation 2) and a parabolic diffusion equation (Equation 3) were adopted [3,27–28] as described by:

[1]
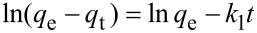


[2]
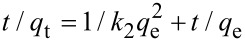


[3]
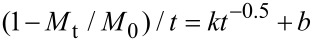


where *q*_e_ and *q*_t_ refer to the released amounts at equilibrium and at time *t* (min), *k* is the equilibrium rate constant, *M*_t_ and *M*_0_ represent the drug content remaining in the SWCNT–COOH at release time 0 and *t*, respectively, and *b* is a constant whose chemical significance is not fully understood.

**Figure 6:**
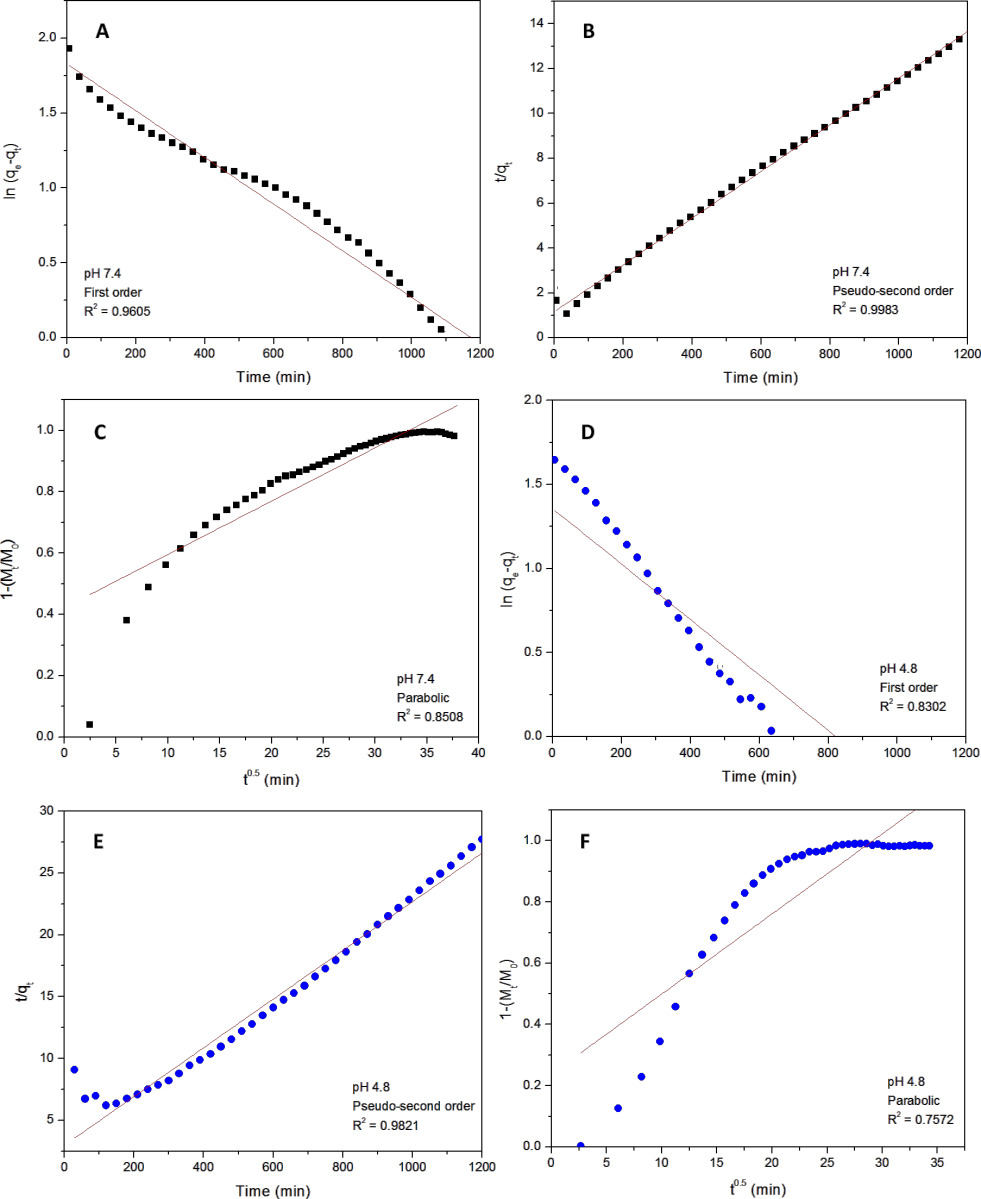
Fitting data for the release of LD from SWCNT–COOH to the pseudo-first-order, pseudo-second-order and the parabolic equation for pH 7.4 (A–C) and pH 4.8 (D–F).

Based on the fitting results of the LD release profiles given in Figure 6, it can be seen that the data conformed best to the pseudo-second-order kinetic model. On the basis of these three models (pseudo-first-order, pseudo-second-order and parabolic diffusion), Figure 6B shows a better fitted release profile at pH 7.4 in PBS solution using the second-order kinetic model resulting in a high correlation coefficient, *R*^2^, of 0.9983. The same kinetic model was also applied to the sample in pH 4.8 PBS solution, resulting in a correlation coefficient of 0.9821 (Figure 6E). The saturated release amount of LD, *R*^2^, rate constant and half-time resulting from the pseudo-second-order model fits are also presented in Table 3. The second-order reaction suggests that the release of LD from its nanocarrier is dependent on the concentration, and that the half-time should increase as the process continues [29]. According to the classification of reaction rates [29], the type of reaction that occurs at pH 7.4 can be considered as “very fast”, whereas the reaction at pH 4.8 is “fast” with a duration of over 1200 min for the complete release of LD. Overall, this experiment shows that the synthesized SWCNT–LD nanohybrid possessed favourable sustained and controlled release properties as a drug carrier.

**Table 3:** Correlation coefficient, rate constant and half-time obtained by fitting the data of the release of LD from SWCNT–COOH into pH 7.4 and pH 4.8 phosphate-buffered saline solutions at 25 °C.

Aqueous solution	Saturated release, [%]	Correlation coefficient, *R*^2^			Rate constant^a^, *k*, [mg/min]	Half-time, *t*_1/2_ [min]

		Pseudo-first- order	Pseudo-second-order	Parabolic diffusion		

pH 7.4	88.6	0.9605	0.9983	0.8508	9.34 × 10^−5^	112
pH 4.8	43.3	0.8302	0.9821	0.7572	1.30 × 10^−4^	151

^a^Estimated using pseudo-second-order kinetics.

### In vitro bioassay

#### PC12 cell lines

PC12 is one of the most widely applied neuronal cell lines and can be used as a model to study secretory activity and catecholamine metabolism and regulation. In this study, we examine the cytotoxic effect of the synthesized SWCNT–LD nanohybrid on PC12 cells using 3-(4,5-dimethylthiazol-2-yl)-2,5-diphenyltetrazolium bromide (MTT). The number of living cells (indicated by the optical density, OD) in each well was directly recorded by the UV absorbance at 24, 48 and 72 h. It is widely believed that the main contributor which induces PD is the degeneration of dopaminergic neurons, and thus, it is crucial to establish a preliminary understanding of the effect of CNTs action on neuronal cells.

**Figure 7:**
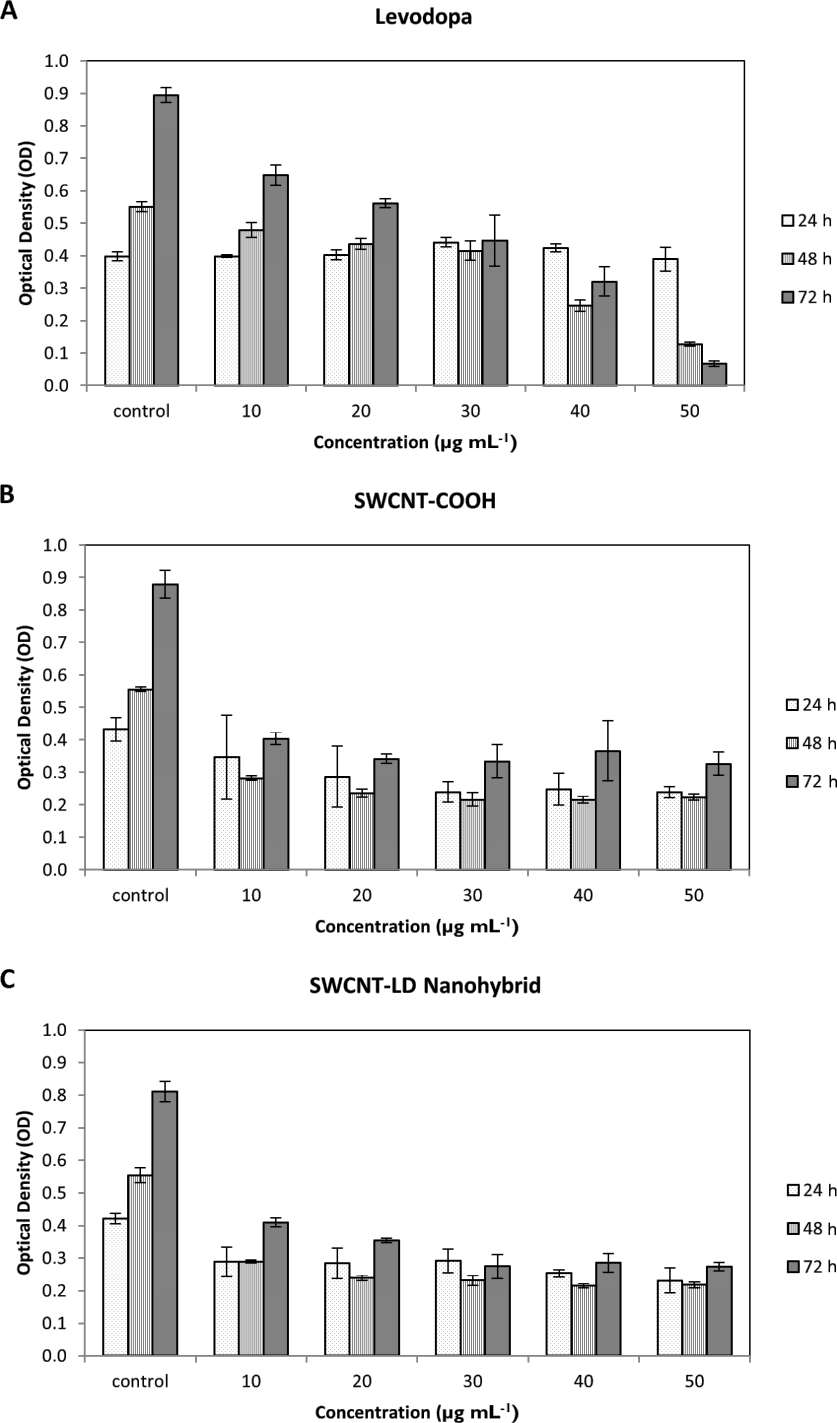
MTT assay of PC12 cell lines after 24, 48 and 72 h of treatment with LD, SWCNT–COOH and SWCNT–LD nanohybrid, at different concentrations. The optical density (OD) is directly correlated to the number of living cells. The data are shown as means ± standard deviation from three individual experiments.

As shown in Figure 7A, we observed a reduction in PC12 cell viability after treatment with the free drug (LD) in a dose- and time-dependent manner at concentrations of 0 μg mL^−1^ (control) to 50 μg mL^−1^. The LD compound demonstrates a sustained decrease in cell viability with increasing concentration at each time point. This observation is comparable with the results published by Mytilineou et al. [30], where the LD exposed to mesencephalic cultures at 72 h of treatment was associated with a dose-dependent reduction in cell survival. The authors attributed the potential toxicity of LD to the decreased antioxidant capacity in the limited environment of the cell culture that makes the neurons more susceptible to LD. In another related work, conducted by Kura et al. [31], viability of PC12 cells was found to decrease with increasing concentration of LD after 72 h, as determined by MTT assay.

In contrast, SWCNT–COOH and SWCNT–LD did not compromise the cell viability of PC12 cells and the OD remains almost constant throughout the experiment after 72 h of treatment at different concentrations (Figure 7B,C). However, the decrease in cell viability of the two compounds as compared to the control may be attributed to the catalyst residue remaining in the tubes as well as the effect of agglomeration of cells with CNTs [32]. This is because CNTs tend to agglomerate into bundle-like clusters due to their hydrophobic surfaces, therefore, the growth of cells can be inhibited by the CNT agglomerates at high concentration. In order to elucidate the cell interaction with the nanohybrid, further cellular uptake experiments are required and are currently under investigation.

## Conclusion

In conclusion, a new, versatile nanohybrid based on a very simple method for the administration of LD has been developed. The findings of this study reveal that the loading capacity of SWCNT–COOH is approximately ≈38.2% as determined by UV–vis spectroscopy. Fourier transform infrared spectra indicated that the LD was successfully conjugated to SWCNT–COOH, and this was further supported by both the CHNS elemental analysis and Raman spectroscopy study. The drug release study shows that the release of LD from SWCNT–COOH is pH-dependent, where the release rate at pH 7.4 in PBS solution is significantly higher than that at pH 4.8. It is also apparent that LD was released in a sustained manner and governed by pseudo-second-order kinetic. The sustained release of LD over more than 20 h demonstrates the potential novelty for further development as a slow, sustained-release formulation. The MTT bioassay results showed a dose-dependent cytotoxicity response from pure LD, whereas SWCNT–COOH and SWCNT–LD did not compromise the viability of PC12 cells, which remained almost constant throughout the experiments. This suggests that the newly synthesized nanohybrid is a promising drug delivery system for the delivery of LD to nervous system.

## Experimental

### Materials

Short, carboxyl single-walled carbon nanotubes (SWCNT–COOH) produced by chemical vapour deposition with a diameter of 1–2 nm an purity of 90% (w/w %) were purchased from Chengdu Organic Chemicals Co., Ltd. (Chengdu, China) and used as received. Pure LD (C_9_H_11_NO_4_, molecular weight 197.19) of 99% purity was purchased from Acros Organics (Geel, Belgium) and used as received. Deionized water was used in all experiments. The rat neuronal cells (PC12) were obtained from American Tissue Culture Collection (ATCC). Dulbecco's Modified Eagle Medium (DMEM), fetal bovine serum (FBS), trypsin-EDTA (1×) and penicillin-streptomycin (100×) were purchased from PAA (Pasching, Austria). 2-(3,5-diphenyltetrazol-2-ium-2-yl)-4,5-dimethyl-1,3-thiazole bromide (MTT) was purchased from PhytoTechnology Laboratories (Kansas, USA). All other reagents and solvents were of analytical grade.

### Preparation of standard solutions

**Figure 8:**
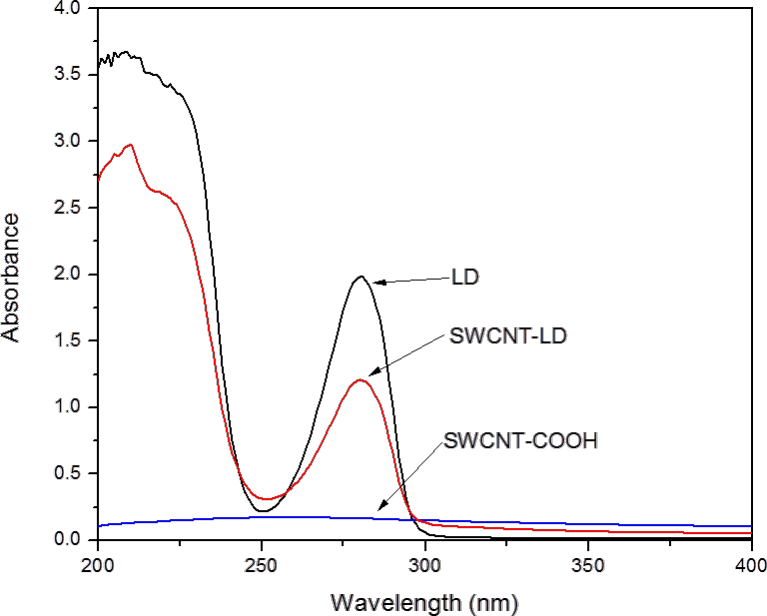
UV–vis absorption spectra of pure LD, supernatant residue and SWCNT–COOH.

A stock solution of LD was prepared by dissolving 8 mg of powder in 16 mL of deionized water. The solution was heated in a water bath at 60 °C for 30 min and then allowed to cool to room temperature for another 30 min. This ensured that the LD was fully dissolved in deionized water. After that, the solution was scanned in range of 200–600 nm to determine the maximum absorption wavelength, λ_max_, of LD (as referenced to a blank reagent) by UV–vis spectroscopy. The λ_max_ of LD was observed to be at 280 nm, as shown in Figure 8. Subsequently, 8 mL of the LD stock solution was then used to prepare a series of standard solutions containing 8 mL of deionized water. The absorbance of the resulting solutions was measured at 280 nm and a calibration curve was plotted to determine the linearity in the range of 0.00–0.125 mg mL^−1^. The linear regression equation obtained from the calibration graph was *y* = 15.5955·*x* (mg mL^−1^) + 0.0843 with a correlation coefficient of 0.9920, where *y* is the absorbance and *x* is the LD concentration.

### Synthesis of SWCNT–LD nanohybrid

Typically, 20 mg of SWCNT–COOH was suspended in deionized water and ultrasonicated for at least 30 min in order to separate the nanotubes from one another. 5 mg of LD was prepared and dissolved completely in 40 mL of deionized water at a concentration of 0.125 mg mL^−1^. Subsequently, this solution was added to the CNT suspension followed by rapid stirring at room temperature in darkness for 24 h. This precaution was taken to prevent light decomposition of the drug. After that, the sample was collected, washed and centrifuged three times at 4000 rpm and dried for 24 h at 60 °C in an oven. The final product was then ground and stored for further use and characterization.

**Scheme 1:**
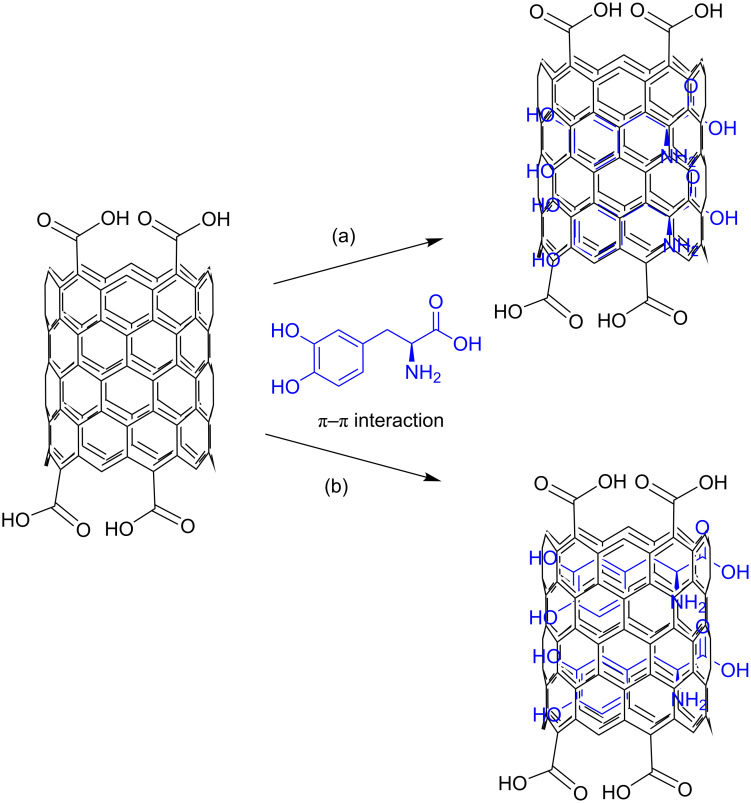
Suggested π–π interaction between LD molecules and carbon nanotubes in a (a) sandwich or (b) parallel-displaced interaction.

The loading capacity of SWCNT–COOH was determined by UV–vis spectroscopy according to a method previously described [2]. After centrifugation, both the solid sample and supernatant residue (unbound LD) were collected. The absorbance of the supernatant residue was measured at 280 nm, which is the characteristic absorbance wavelength of LD (Figure 8). By comparing the absorbance value of the residue with the free LD solution, the loading capacity of the SWCNT–COOH was estimated to be about 38.2%. According to Wheeler [33], there are two major paths for noncovalent π-stacking interactions involving aromatic rings to occur: sandwich and parallel-displaced. Sandwich is a π-stacking geometry that occurs between the benzene groups through electrostatic repulsion, whereas the electrostatic interaction between the benzenes will result in a parallel-displaced geometry. A schematic representation of the possible noncovalent interaction (a) sandwich or (b) parallel-displaced, between LD molecules and SWCNT–COOH is given in Scheme 1.

### In vitro drug release response

The drug release mechanisms of LD from SWCNT–COOH were studied at room temperature using two different pH values, namely a pH value of 7.4 and 4.8 in phosphate buffered saline (PBS) solutions, based on a method previously described [34–35]. A pH of 7.4 was chosen to demonstrate the drug release of LD in physiological environment, whereas a pH of 4.8 mimics the acidic conditions of the human stomach after food consumption. Approximately 11.43 mg of the sample was added to 40 mL of the medium and the accumulated amount of LD released was measured at 280 nm using a UV–vis spectrophotometer.

### Characterization

UV–vis spectroscopy was used to study the loading capacity and controlled release properties of the material using a Lambda 35 spectrophotometer (Perkin Elmer, Boston, MA). In order to study the functional groups present in the materials, Fourier transform infrared (FTIR) spectra of the samples were recorded over the range of 4000–500 cm^−1^ on a 1752X FTIR (Perkin Elmer, Waltham, MA) using a KBr disc. For carbon, hydrogen, nitrogen and sulfur (CHNS) analysis, a CHNS-932 from LECO Instrument (St Joseph, MI) was used. Raman spectra were collected using a UHTS 300 Raman spectrometer (WITec, Germany) with an excitation wavelength at 532 nm. CNT samples were deposited on glass slides and detailed scans were performed in the 100–2000 cm^−1^ range. The nanotube product was first ultrasonicated in ethanol using a PowerSonic 420 (Hwashin Technology Co., Korea) device. Several droplets of the nanotube suspension were deposited onto a glass slide and then air dried at room temperature. The surface morphology changes of the carbon samples before and after drug loading were observed with a field emission scanning electron microscope (FESEM). The samples were sputter-coated with gold and examined in a JSM-7600F SEM (JEOL, Japan). The internal structure of the nanotubes was observed on a transmission electron microscope (TEM), Tecnai G2 (FEI, USA). The samples were prepared by placing a drop of a sonicated dispersion on the carbon grid and dried at 37 °C for 24 h.

### PC12 cell lines

The cells were grown in DMEM and supplemented with 10% FBS, penicillin (100 mg/mL) and streptomycin (100 mg/mL), and incubated at 37 °C in a humidiﬁed chamber, 5% CO_2_ atmosphere. The MTT colorimetric assay was carried out as described previously [36]. Brieﬂy, PC12 cells were seeded in 96-well, flat-bottomed plates with 5000 cells per well and incubated at 37 °C (5% CO_2_ and 95% air) for 24 h to allow cell attachment. Subsequently, the cells were treated with different concentrations of LD, SWCNT–COOH and SWCNT–LD for 24, 48 and 72 h. Following incubation, 20 µL MTT (5 mg/mL in PBS) was added to each well and the plate was incubated for 3 h. The excess MTT was then aspirated and the formazan crystals formed were dissolved with 150 µL of DMSO. The optical density (OD), which was proportional to cell viability, was measured with a spectrophotometer at 570 nm with a reference wavelength of 630 nm. Experiments were performed in triplicate and the results were expressed as mean ± standard deviation.
